# Supplementary figures and images for: Exploring Novel Biomarkers for an Acute Coronary Syndrome Diagnosis Utilizing Plasma Metabolomics
Source: Int J Mol Sci. 2024 Jun 18;25(12):6674. doi: 10.3390/ijms25126674 (PMC11204280; doi:10.3390/ijms25126674)

## Slide 1
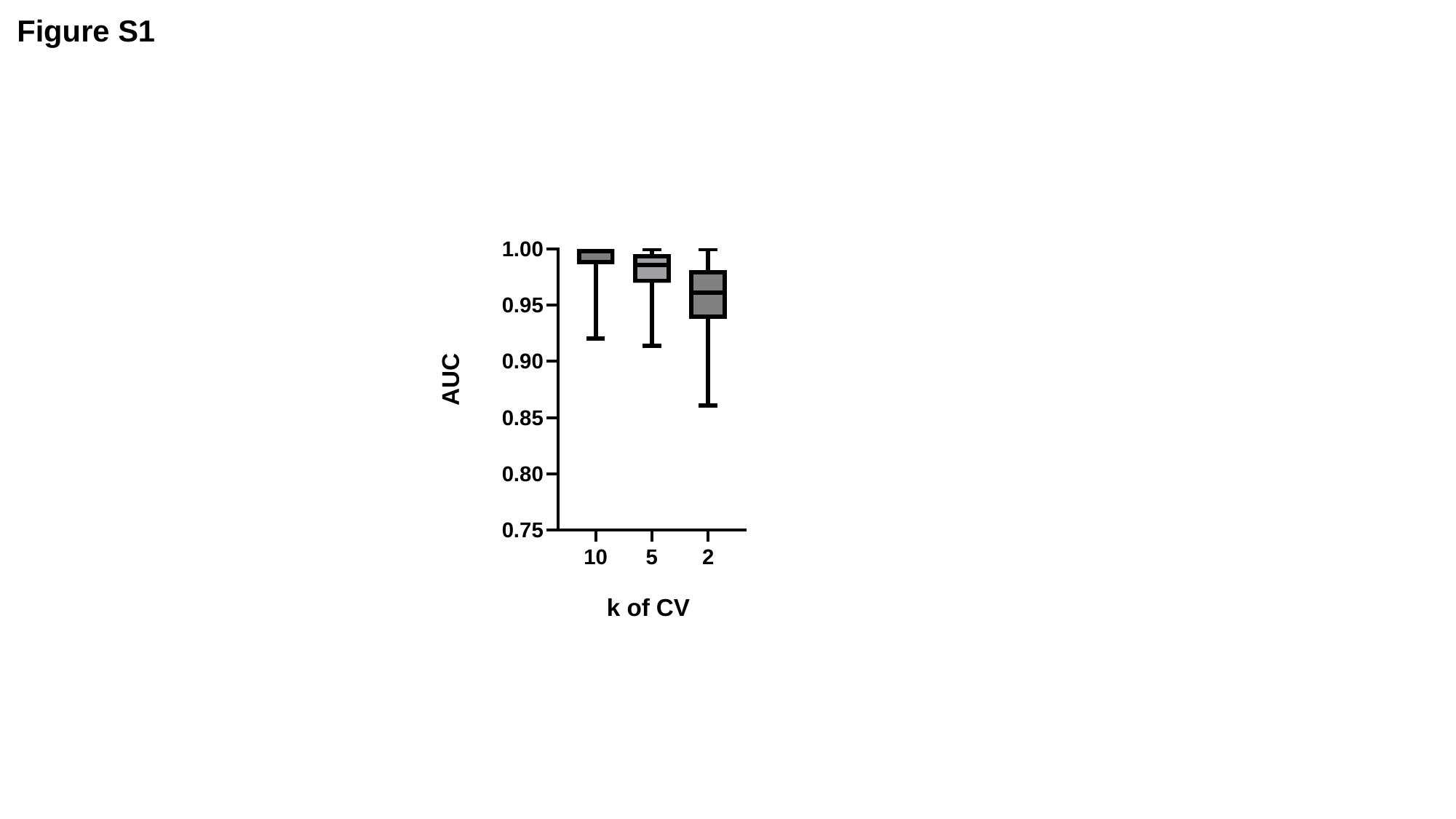

Figure S1
AUC
k of CV

Supplement: Supplementary file 1 [file ijms-25-06674-s001.zip › ijms-3022894-supplementary.pptx]
